# A British Hospital at Limoges

**Published:** 1915-11-27

**Authors:** Lindsey Smith

**Affiliations:** Hon. Secretary.


### THE EDITOR'S LETTER-BOX.

### "A BRITISH HOSPITAL AT LIMOGES."

*To the Editor of* The Hospital.

Sir,—The attention of the Wounded Allies Relief Committee has been drawn to your paragraphs of last week referring to their hospital at Limoges. My Committee think it due to Dr. Brend and to themselves to send you the following comments, which they must ask you to publish.

The extracts from Dr. Brend's report which were published in the Press were issued without the knowledge of the Committee or Dr. Brend, and convey a wrong impression of the position of this hospital. Although fully equipped, the hospital, which has been in full swing for over a year, is bound to require renewal of equipment in various directions. To obtain this assistance was probably the intention of the too enthusiastic contributor who sent the unauthorised paragraph which appeared recently in the Press.

You say of such hospitals, "They are always small, therefore inefficient." If this is true of a hospital of 170 beds, it applies to a large number of British and Red Cross hospitals. As a matter of fact it is not true, for this number of beds is quite sufficient for economic working. You further say, "They cannot be, and are not, in fact, provided with adequate special departments in the way of *x*-rays, bacteriology, and so forth." This hospital, as is the case with all our hospitals, possesses a complete *x*-ray installation. All bacteriological investigations required are performed by experts under the French authorities.

As regards your statement that the hospital is "working at a vast distance from the firing-line," permit me to inform you that the wounded French soldiers reach our hospital within thirty-six hours of being wounded. The French have hospitals much further from the firing-line than Limoges, and we ourselves have hospitals as far north at least as Edinburgh and Glasgow.

The suggestion that the equipment and staff could be better employed for our own troops cannot be substantiated by the facts. The Wounded Allies Relief Committee have always endeavoured not to compete with British medical needs. A considerable proportion of the medical staffs in our hospitals consist of women doctors. Several of our male doctors have been refused by the R.A.M.C. on the ground of physical defects or age, which, however, in no way interfered with their medical capacity, and two are Belgians. A considerable proportion of our nurses, while competent and highly skilled, are ineligible for various reasons to join British units. An appreciable part of our funds has been contributed by Americans and Belgians, and would not therefore have been available for British military forces.

The words "isolated and inco-ordinated enterprises" are not justified when applied to work on the scale of that undertaken by the Wounded Allies Relief Committee. The Committee is recognised and assisted by the British, French, and Belgian Governments in its many activities, of which the French hospitals are only a small part.

With regard to your comments on Dr. Brend, who has given us his invaluable services for more than a year, it is sufficient to say that they can only have been written by a person unaware of his qualifications and experience. The paragraphs in The Hospital have been written by someone who has presumably not had an opportunity of seeing for himself the state of affairs in France. There is every reason to think now that there are sufficient doctors with the English troops, but, owing to the enormously large number of wounded in the French Army, and in spite of the brilliant efforts of the French medical authorities and the devotion of all classes of the community, many hospitals in France are still in need of doctors, nurses, and equipment, owing to obvious reasons. We owe it to our Allies to redouble the efforts which are being made in this country, rather than to "crab" them by ill-considered and uninformed statements.—Yours faithfully,

Lindsey Smith, Hon. Secretary.

November 23, 1915.

[We are glad to print this expression of opinion, though we are not convinced by the arguments adduced. For instance, Sir Lindsey Smith assures us that the hospital at Limoges possesses an *x*-ray installation, though he does not say whether an expert radiologist is in charge of it. Our point is that both a radiologist and an installation are partly wasted in so small a hospital; one plant and one operator can do the work for a very much larger hospital, and this is a good example of the wastefulness of all small war hospitals. It is precisely because our contributor has not merely seen the working of both large and small hospitals in France, but has himself taken part in administration there, that we attach some importance to his views; but the principle at stake obviously admits of differences of opinion. A further reference to this matter will be found on another page.—Ed. The Hospital.]

